# Feces Metagenomes and Metagenome-Assembled Genome Sequences from Two Separate Dogs (Canis lupus familiaris) with Multiple Diarrheal Episodes

**DOI:** 10.1128/MRA.01065-20

**Published:** 2020-11-25

**Authors:** Tshepiso Pleasure Ateba, Kazeem Adekunle Alayande, Mulunda Mwanza

**Affiliations:** a Department of Animal Health, Faculty of Natural and Agricultural Sciences, Mafikeng Campus, North West University, Mmabatho, Mafikeng, South Africa; b Department of Microbiology, Faculty of Natural and Agricultural Sciences, Mafikeng Campus, North West University, Mmabatho, Mafikeng, South Africa; University of Maryland School of Medicine

## Abstract

This study reports two feces metagenomes (D84 and D85) and six metagenome-assembled genomes (MAGs). The assembled MAGs include *Pseudomonas* sp. strain NID84 and Acinetobacter sp. strain N2D84 from D84 and *Enterococcus* sp. strain N4D85, *Enterococcus* sp. strain N5D85, *Lactobacillus* sp. strain N6D85, and *Leuconostoc* sp. strain N7D85 from D85. Acinetobacter sp. N2D84 was identified as a human pathogen with a probability of 92%.

## Abstract (precis)

This study reports two feces metagenomes (D84 and D85) and six metagenome-assembled genomes (MAGs). The assembled MAGs include *Pseudomonas* sp. strain NID84 and Acinetobacter sp. strain N2D84 from D84 and *Enterococcus* sp. strain N4D85, *Enterococcus* sp. strain N5D85, *Lactobacillus* sp. strain N6D85, and *Leuconostoc* sp. strain N7D85 from D85. Acinetobacter sp. strain N2D84 was identified as a human pathogen with a probability of 92%.

## ANNOUNCEMENT

Gastroenteritis is a common disease in dogs caused by a number of microorganisms, including bacteria and viruses. Its symptoms vary from diarrhea and abdominal pain to acute hemorrhagic syndrome, and it can be fatal (1).

Fecal samples were separately collected from two diarrhea-affected dogs (Canis lupus familiaris) that were housed by the Society for the Prevention of Cruelty to Animals (SPCA) in Mafikeng, North West Province, South Africa. The samples were collected directly from the rectum with sterile gloves and immediately placed in sterile containers. Metagenomic DNA was extracted from 150 mg of the fecal sample using a Quick-DNA fecal/soil microbe miniprep kit (Zymo Research Corp., USA) following the manufacturer’s instruction. The library was prepared with a Nextera DNA Flex library prep kit using Nextera DNA CD index adapters (96 indexes plated) and sequenced on an Illumina NovaSeq instrument. A total of 12,512,020 reads were generated from sample D84 and 19,928,158 from sample D85, both with 1 × 150-bp paired-end read lengths, while the depth of sequencing for D84 was 156× and that for D85 was 153×.

The metagenomic data were filtered for adapter regions and low-quality reads using Trimmomatic v0.36 (2). The quality reads were taxonomically classified using Kaiju v1.7.2 (3) and GOTTCHA2 v2.1.6 and thereafter assembled using metaSPAdes v3.13.0 (4) and MEGAHIT v1.2.9 (5) for samples D84 and D85, respectively. Each assembly was binned into metagenome-assembled genomes (MAGs) using MaxBin2 v2.2.4 (6). The quality and completeness of each MAG was assessed using CheckM v1.0.18 (7), and redundant bins were excluded from further analysis. The taxonomic assignments were obtained for the MAGs based on the genome taxonomy database using GTDB-Tk v1.1.0 (8).

The MAGs were annotated using the NCBI Prokaryotic Genome Annotation Pipeline (PGAP) v4.12 (9). The extent of pathogenicity and acquired drug-resistant genes were determined for the MAGs using PathogenFinder v1.1 (10) and ResFinder v4.0, respectively (11). Most of the software was accessed through the KBase workspace service v0.11.1 (12), and default parameters were used for all the software employed in the analysis.

The assembly size of the feces metagenome from sample D84 is 10,293,073 bp, and it contains 575 contigs with a G+C content of 52.23%, an *N*_50_ value of 52,139 bp, and an *L*_50_ value of 37. The entire assembly, which contains 7,693 coding sequences, was binned into two MAGs, which were identified as *Pseudomonas* sp. strain NID84 and Acinetobacter sp. strain N2D84 (Table 1). As for sample D85, the assembly size is 16,848,868 bp, distributed in 2,968 contigs, and it has an average G+C content of 37.35%. The *N*_50_ value of the assembly is 7,085 bp, while the *L*_50_ value is 570, and the entire metagenome contains 8,163 predicted genes. Four different MAGs were extracted from the metagenome and identified as *Enterococcus* sp. strain N4D85, *Enterococcus* sp. strain N5D85, *Lactobacillus* sp. strain N6D85, and *Leuconostoc* sp. strain N7D85 (Table 1). Not all the contigs contained the MAGs in both samples, and the unbinned contigs were discarded. Acinetobacter sp. N2D84 was identified as a human pathogen with a probability of 92% and 13 matched pathogenic families.

**TABLE 1:** Features and accession numbers of the metagenome-assembled genomes from the feces metagenomes^*a*^

Physical sample	MAG identity	Genome size (bp)	Total no. of CDS^*b*^	No. of contigs	*N*_50_ (bp)	G+C content (%)	CMP^*c*^ (%)	CNT^*d*^ (%)	GenBank accession no.
D84	*Pseudomonas* sp. NID84	6,374,370	5,798	168	98,074	59.00	99.68	4.06	JACRYQ000000000
	Acinetobacter sp. N2D84	3,755,466	3,729	373	11,956	41.17	94.72	10.15	JACRYP000000000
D85	*Enterococcus* sp. N4D85	3,185,421	3,438	678	5,355	37.72	76.84	21.84	JACUTJ000000000
	*Enterococcus* sp. N5D85	4,273,788	4,421	616	9,391	39.29	75.78	33.33	JACUTK000000000
	*Lactobacillus* sp. N6D85	3,558,646	3,945	592	8,543	34.23	67.24	14.37	JACUTL000000000
	*Leuconostoc* sp. N7D85	2,401,161	2,582	445	5,665	39.19	87.69	38.16	JACUTM000000000

a The values for completeness and contamination of each MAG were determined using CheckM v1.0.18. The genome sizes were determined using v1-KBaseGenomeAnnotations.Assembly-5.0 and NCBI Prokaryotic Genome Annotation Pipeline (PGAP).

b CDS, coding sequences.

c CMP, completeness.

d CNT, contamination.

Ethical clearance for the study was approved by the Research Ethics Committee of the North West University, South Africa (NWU-00160-14-A9).

### Data availability.

All data were deposited under the GenBank BioProject number PRJNA655841. These whole-genome shotgun projects have been deposited in DDBJ/ENA/GenBank under the accession numbers JACRYN000000000 and JACRYO000000000. The versions described in this paper are the first versions, JACRYN010000000 and JACRYO010000000. The SRA accession numbers for the raw reads are SRX8905271 and SRX8949278 for samples D84 and D85, respectively.
